# Supplementary material for: Phage Resistance Modulates Escherichia coli B Response to Metal-Based Antimicrobials
Source: Antibiotics (Basel). 2025 Sep 18;14(9):942. doi: 10.3390/antibiotics14090942 (PMC12466345; doi:10.3390/antibiotics14090942)
Supplement: Supplementary file 1 [file antibiotics-14-00942-s001.zip › antibiotics-3860120-supplementary.pdf]

Supplementary

Supplementary Table S1. Significant Polymorphisms in Ancestor, Control, Phage-selected, Iron(III)-selected and Phage/ iron(III)-selected populations

		**Ancestors populations**									
**Gene**	**Position**	**ANC$_1$**	**ANC$_2$**	**ANC$_3$**	**ANC$_4$**	**ANC$_5$**	**ANC$_6$**	**ANC$_7$**	**ANC$_8$**	**ANC$_9$**	**ANC$_{10}$**
pgap_annot_001232 →	1224879	0.400	0.915	0.000	0.000	0.000	0.395	0.442	0.489	0.462	0.394
rhsC ←	1406366	0.245	0.440	0.423	0.427	0.502	0.192	0.131	0.000	0.116	0.118
mqo → / ← eco	4486987	0.140	0.529	0.563	0.558	0.642	0.000	0.000	0.142	0.174	0.000
		**Control Population**									
**Gene**	**Position**	**Ctrl$_1$**	**Ctrl$_2$**	**Ctrl$_3$**	**Ctrl$_4$**	**Ctrl$_5$**	**Ctrl$_6$**	**Ctrl$_7$**	**Ctrl$_8$**	**Ctrl$_9$**	**Ctrl$_{10}$**
rhsC ←	1406366	0.417	0.000	0.461	0.394	0.443	0.450	0.424	0.314	0.421	0.360
pgap_annot_002794 ←	2834658	0.000	0.000	0.399	0.000	0.000	0.000	0.000	0	0	0.000
pgap_annot_002794 ←	2834676	0.000	0.000	0.450	0.000	0.000	0.000	0.000	0	0	0.000
pgap_annot_002794 ←	2834686	0.000	0.000	0.500	0.000	0.000	0.000	0.000	0	0	0.000
pgap_annot_003814 ←	3901227	0.000	0.000	0.463	0.000	0.000	0.000	0.000	0	0	0.000
pgap_annot_003814 ←	3901239	0.000	0.000	0.502	0.000	0.000	0.000	0.000	0	0	0.000
pgap_annot_003814 ←	3901240	0.000	0.000	0.503	0.000	0.000	0.000	0.000	0	0	0.000
pgap_annot_003814 ←	3901273	0.000	0.000	0.366	0.000	0.000	0.000	0.000	0	0	0.000
pgap_annot_003814 ←	3901274	0.000	0.000	0.366	0.000	0.000	0.000	0.000	0	0	0.000
pgap_annot_003814 ←	3901275	0.000	0.000	0.366	0.000	0.000	0.000	0.000	0	0	0.000
mqo → / ← eco	4486987	0.619	0.000	0.269	0.643	0.531	0.607	0.613	0.276	0.459	0.000
		**Phage-selected population**									
**Gene**	**Position**	**PS$_1$**	**PS$_2$**	**PS$_3$**	**PS$_4$**	**PS$_5$**	**PS$_6$**	**PS$_7$**	**PS$_8$**	**PS$_9$**	**PS$_{10}$**
asmA →	44559	0.000	0.000	0.000	0.000	0.000	0.000	0.000	0.670	0.000	0.000
ompA →	1081880	0.000	0.552	0.150	0.000	0.000	0.000	0.000	0.000	0.000	0.000
pgap_annot_001232 →	1224879	0.000	0.831	0.232	0.000	0.000	0.000	0.000	0.000	0.000	0.000
rhsC ←	1406366	0.420	0.421	0.435	0.435	0.441	0.395	0.475	0.512	0.460	0.449
arcA →	2120517	0.618	0.000	0.000	0.000	0.000	0.000	0.000	0.000	0.000	0.000
pgap_annot_002794 ←	2834658	0.000	0.000	0.000	0.000	0.000	0.000	0.000	0.000	0.827	0.000
pgap_annot_002794 ←	2834676	0.000	0.000	0.000	0.000	0.000	0.000	0.000	0.000	0.836	0.000
pgap_annot_002794 ←	2834686	0.000	0.000	0.000	0.000	0.000	0.000	0.000	0.000	0.844	0.000
waaA ← / → rfaQ	2996757	0.000	0.000	0.000	0.000	0.000	0.000	0.669	0.000	0.000	0.947
waaG →	2998655	0.000	0.000	0.000	0.000	0.000	0.000	0.674	0.000	0.000	0.000
		**Iron(III)-selected Populations**									
**Gene**	**Position**	**Fe$_1$**	**Fe$_2$**	**Fe$_3$**	**Fe$_4$**	**Fe5**	**Fe$_6$**	**Fe$_7$**	**Fe$_8$**	**Fe$_9$**	
rhsC ←	1406366	0.000	0.000	0.000	0.000	0.442	0.471	0.468	0.422	0.000	
basR →	2436088	0.000	0.336	0.662	0.000	0.000	0.000		0.000	0.000	
rpoC ←	2577900	0.000	0.000	0.000	0.000	0.261	0.186	0.565	0.211	0.000	
dctA →	3131445	0.000	0.000	0.000	0.938	0.000	0.000	0.000	0.000	0.173	
aroK →	3295699	0.000	0.576	0.886	0.000	0.528	0.927	0.948	0.375	0.218	
qseB ←	3638856	0.000	0.000	0.000	0.942	0.000	0.000	0.000	0.000	0.316	
rseB →	4111967	0.000	0.531	0.000	0.000	0.000	0.000	0.000	0.000	0.000	
rseB →	4112525	0.000	0.000	0.767	0.000	0.000	0.000	0.000	0.000	0.104	
mqo → / ← eco	4486987	0.513	0.522	0.536	0.507	0.000	0.000	0.000	0.000	0.000	
qseB ←	3639008	0.649	0.000	0.000	0.000	0.000					
		**Phage/iron(III)-selected Populations**									
**Gene**	**Position**	**P/f$_1$**	**P/f$_2$**	**P/f$_3$**	**P/f$_4$**	**P/f$_5$**	**P/f$_6$**	**P/f$_7$**	**P/f$_8$**	**P/f$_9$**	**P/f$_{10}$**
infC →	341288	0.000	0.000	0.000	0.438	0.055	0.151	0.000	0.000	0.000	0.000
rhsC ←	1406366	0.538	0.451	0.433	0.439	0.397	0.450	0.425	0.508	0.425	0.466
basR →	2436088	0.000	0.233	0.000	0.000	0.000	0.000	0.172	0.000	0.151	0.000
basR →	2436120	0.346	0.225	0.000	0.000	0.000	0.000	0.000	0.000	0.000	0.000
basR →	2436238	0.485	0.000	0.000	0.000	0.000	0.000	0.000	0.000	0.000	0.000
basR →	2436523	0.000	0.000	0.000	0.000	0.348	0.000	0.000	0.000	0.694	0.000
basR →	2436573	0.000	0.000	0.911	0.000	0.000	0.351	0.559	0.000	0.000	0.894
rpoC ←	2577900	0.000	0.354	0.000	0.365	0.487	0.000	0.000	0.000	0.000	0.000
pgap_annot_003609 →	3678916	0.000	0.000	0.348	0.000	0.000	0.000	0.278	0.000	0.000	0.000
rseB →	4111967	0.343	0.439	0.000	0.000	0.000	0.144	0.000	0.000	0.000	0.000
menH →	4418228	0.000	0.000	0.000	0.391	0.073	0.000	0.474	0.569	0.541	0.539

Supplementary Table S2: Population, Gene, Mutation, Annotation, and Description

	Gene	Mutation	Annotation	Description
**Ancestor**	pgap_annot_001232 →	A→G	pseudogene (534/1545 nt)	rhs element protein RhsC
	rhsC ←	G→T	E43* (GAG→TAG)	2-dehydropantoate 2-reductase
	mqo → / ← eco	G→T	Q221K (CAA→AAA)	autotransporter outer membrane beta-barrel domain-containing protein
**Control**	rhsC ←	A→G	pseudogene (534/1545 nt)	rhs element protein RhsC
	pgap_annot_002794 ←	C→T	noncoding (45/77 nt)	tRNA-Asp
	pgap_annot_002794 ←	T→C	noncoding (27/77 nt)	tRNA-Asp
	pgap_annot_002794 ←	G→A	noncoding (17/77 nt)	tRNA-Asp
	pgap_annot_003814 ←	C→A	noncoding (64/77 nt)	tRNA-Met
	pgap_annot_003814 ←	G→T	noncoding (52/77 nt)	tRNA-Met
	pgap_annot_003814 ←	A→G	noncoding (51/77 nt)	tRNA-Met
	pgap_annot_003814 ←	A→G	noncoding (18/77 nt)	tRNA-Met
	pgap_annot_003814 ←	G→A	noncoding (17/77 nt)	tRNA-Met
	pgap_annot_003814 ←	G→A	noncoding (16/77 nt)	tRNA-Met
	mqo → / ← eco	A→G	intergenic (+348/+461)	malate dehydrogenase (quinone)/serine protease inhibitor ecotin
**Phage-selected**	asmA →	C→G	T168R (ACA→AGA)	outer membrane assembly protein AsmA
	ompA →	A→C	T51P (ACC→CCC)	porin OmpA
	pgap_annot_001232 →	G→C	E252Q (GAA→CAA)	terminase ATPase subunit family protein
	rhsC ←	A→G	pseudogene (534/1545 nt)	rhs element protein RhsC
	arcA →	A→C	D99A (GAC→GCC)	two-component system response regulator ArcA
	pgap_annot_002794 ←	C→T	noncoding (45/77 nt)	tRNA-Asp
	pgap_annot_002794 ←	T→C	noncoding (27/77 nt)	tRNA-Asp
	pgap_annot_002794 ←	G→A	noncoding (17/77 nt)	tRNA-Asp
	waaA ← / → rfaQ	C→T	intergenic (-273/-140)	lipid IV(A) 3-deoxy-D-manno-octulosonic acid transferase/ lipopolysaccharide core heptosyltransferase RfaQ
	waaG →	Δ1 bp	coding (704/1125 nt)	glycosyltransferase family 4 protein
	alaS → / → csrA	G→T	intergenic (+180/-55)	alanine--tRNA ligase/carbon storage regulator CsrA
	pgap_annot_004003 →	G→A	noncoding (895/1542 nt)	16S ribosomal RNA
	pgap_annot_004003 →	A→G	noncoding (906/1542 nt)	16S ribosomal RNA
	aegA →	A→T	N83I (AAT→ATT)	formate-dependent uric acid utilization protein AegA
	mqo → / ← eco	A→G	intergenic (+348/+461)	malate dehydrogenase (quinone)/serine protease inhibitor ecotin
**Iron(III)-selected**	rhsC ←	A→G	pseudogene (534/1545 nt)	
	basR →	C→T	T22I (ACC→ATC)	two-component system response regulator BasR
	basR →	C→A	L99M (CTG→ATG)	two-component system response regulator BasR
	rpoC ←	C→T	E175K (GAA→AAA)	
	dctA →	T→C	F336L (TTT→CTT)	C4-dicarboxylate transporter DctC
	aroK →	C→T	Q23* (CAG→TAG)	shikimate kinase AroK
	qseB ←	C→T	E175K (GAA→AAA)	two-component system response regulator QseB
	rseB →	T→C	L122P (CTT→CCT)	sigma-E factor regulatory protein RseB
	rseB →	T→C	I308T (ATT→ACT)	sigma-E factor regulatory protein RseB
	mqo → / ← eco	A→G	intergenic (+348/+461)	malate dehydrogenase (quinone)/serine protease inhibitor ecotin
	qseB ←	Ins*	coding (371/660 nt)	two-component system response regulator QseB
**Phage/iron(III)-selected**	rhsC ←	A→G	pseudogene (534/1545 nt)	rhs element protein RhsC
	basR →	C→T	T22I (ACC→ATC)	two-component system response regulator BasR
	basR →	G→T	A33S (GCG→TCG)	two-component system response regulator BasR
	basR →	C→T	T72I (ACC→ATC)	two-component system response regulator BasR
	basR →	G→A	E184K (GAA→AAA)	two-component system response regulator BasR
	rpoC ←	C→T	E175K (GAA→AAA)	DNA-directed RNA polymerase subunit beta'
	pgap_annot_003609 →	C→T	A256V (GCG→GTG)	dTMP kinase
	rseB →	T→C	L122P (CTT→CCT)	sigma-E factor regulatory protein RseB
	menH →	C→T	R161R (CGC→CGT)	2-succinyl-6-hydroxy-2, 4-cyclohexadiene-1-carboxylate synthase
	mqo → / ← eco	A→G	intergenic (+348/+461)	malate dehydrogenase (quinone)/serine protease inhibitor ecotin

Ins* = (TACCGTGGCGCAGCTCGTTGC)1→2, referring to a duplication of this sequence beginning at position 3,639,008.

Supplementary Table S3. Selective Sweeps in Ancestor, Control, Phage-selected, Iron(III)-selected and Phage/ iron(III)-selected populations

		**Ancestor Populations**									
**Gene**	**Position**	**ANC$_1$**	**ANC$_2$**	**ANC$_3$**	**ANC$_4$**	**ANC$_5$**	**ANC$_6$**	**ANC$_7$**	**ANC8**	**ANC9**	**ANC10**
hchA ←	144,564	0.000	1.000	1.000	1.000	1.000	0.000	0.000	0.000	0.000	0.000
lon ← / → pgap_annot_001702	1,688,040	0.000	1.000	1.000	1.000	1.000	0.000	0.000	0.000	0.000	0.000
cynR →	1,787,666	0.000	0.000	0.000	0.000	0.000	0.000	0.000	1.000	0.000	0.000
cynR →	1,787,673	0.000	0.000	0.000	0.000	0.000	0.000	0.000	1.000	0.000	0.000
dcuS →	2,419,167	0.000	0.000	0.000	0.000	0.000	0.000	0.000	1.000	0.000	0.000
pmrB →	2,437,579	1.000	1.000	1.000	1.000	1.000	1.000	1.000	0.000	1.000	1.000
lhgO ←	4,056,974	0.000	1.000	1.000	1.000	1.000	0.000	0.000	0.000	0.000	0.000
pgap_annot_004004 → / → 004005	4,091,013	1.000	1.000	1.000	1.000	1.000	1.000	1.000	1.000	1.000	1.000
pgap_annot_004004 → / → 004005	4,091,016	1.000	1.000	1.000	1.000	1.000	1.000	1.000	1.000	1.000	1.000
pgap_annot_004004 → / → 004005	4,091,109	1.000	1.000	1.000	1.000	1.000	1.000	1.000	1.000	1.000	1.000
pgap_annot_004004 → / → 004005	4,091,112	1.000	1.000	1.000	1.000	1.000	1.000	1.000	1.000	1.000	1.000
pgap_annot_004004 → / → 004005	4,091,145	1.000	1.000	1.000	1.000	1.000	1.000	1.000	1.000	1.000	1.000
mqo → / ← eco	4,487,064	1.000	1.000	1.000	1.000	1.000	1.000	1.000	1.000	1.000	1.000
mqo → / ← eco	4,487,069	1.000	1.000	1.000	1.000	1.000	1.000	1.000	1.000	1.000	1.000
		**Control Populations**									
**Gene**	**Position**	**Ctrl$_1$**	**Ctrl$_2$**	**Ctrl$_3$**	**Ctrl$_4$**	**Ctrl$_5$**	**Ctrl$_6$**	**Ctrl$_7$**	**Ctrl$_8$**	**Ctrl$_9$**	**Ctrl$_{10}$**
pgap_annot_001232 →	1224879	0.000	0.000	1.000	0.000	0.000	0.000	0.000	0.000	0.000	0.000
		**Phage-selected Population**									
**Gene**	**Position**	**PS$_1$**	**PS$_2$**	**PS$_3$**	**PS$_4$**	**PS$_5$**	**PS$_6$**	**PS$_7$**	**PS$_8$**	**PS$_9$**	**PS$_{10}$**
waaA ← / → rfaQ	2996759	0.000	0.000	0.000	0.000	0.000	0.000	0.137	0.000	1.000	0.000
waaG →	2998755	0.000	0.000	0.000	0.000	1.000	0.000	0.000	0.000	0.000	0.000
waaG →	2998967	0.000	0.000	0.000	0.000	0.000	0.000	0.000	0.000	1.000	0.000
[pgap_annot_003642]–003643	3718584	1.000	0.000	0.000	0.000	0.000	0.000	0.000	0.000	1.000	0.000
pgap_annot_003654–003666	3731988	0.000	0.000	0.000	0.000	0.000	0.000	0.000	0.000	1.000	0.000
		**Iron(III)-selected populations**									
**Gene**	**Position**	**Fe$_1$**	**Fe$_2$**	**Fe$_3$**	**Fe$_4$**	**Fe$_5$**	**Fe$_6$**	**Fe$_7$**	**Fe$_8$**	**Fe$_9$**	
ydbD ←	674361	0.000	0.000	0.000	1.000	0.000	0.000	0.000	0.000	0.150	
basR →	2436318	0.000	0.577	0.942	0.069	0.522	0.933	1.000	0.458	0.154	
fieF ← / ← cpxP	2657456	1.000	0.380	0.000	0.000	0.000	0.000	0.000	0.000	0.000	
qseC ←	3637827	0.000	0.000	0.000	1.000	0.000	0.000	0.000	0.000	0.000	
qseB ←	3639336	1.000	0.396	0.068	0.000	0.000	0.000	0.000	0.000	0.000	
		**Phage/iron(III)-selected populations**									
**Gene**	**Position**	p/f$_1$	p/f$_2$	p/f$_3$	p/f$_4$	p/f$_5$	p/f$_6$	p/f$_7$	p/f$_8$	p/f$_9$	p/f$_{10}$
basR →	2436318	1.000	1.000	1.000	0.898	1.000	0.786	1.000	1.000	1.000	1.000
aroK →	3295699	1.000	1.000	1.000	1.000	1.000	0.772	1.000	1.000	1.000	1.000
rseB →	4112525	0.493	0.077	0.000	0.000	0.000	0.000	0.067	1.000	0.221	0.000

Supplementary Table S4 : Population, Gene, Mutation, Annotation, and Description

Populations	Gene	Mutation	Annotation	Description
**Ancestors**	hchA ←	C→A	pseudogene (271/645 nt)	protein deglycase
	lon ← / → pgap_annot_001702	G→A	intergenic (-108/-48)	endopeptidase La/IS4-like element IS421 family transposase
	cynR →	C→T	A13V (GCT→GTT)	transcriptional regulator CynR
	cynR →	T→C	H15H (CAT→CAC)	transcriptional regulator CynR
	dcuS →	Δ5 bp	pseudogene (862-866/1637 nt)	sensor histidine kinase
	pmrB →	G→T	G296V (GGA→GTA)	two-component system sensor histidine kinase PmrB
	lhgO ←	G→A	A72V (GCC→GTC)	L-2-hydroxyglutarate oxidase
	pgap_annot_004004 → / → 004005	G→A	intergenic (+25/-160)	tRNA-Glu/23S ribosomal RNA
	pgap_annot_004004 → / → 004005	2 bp→TG	intergenic (+28/-156)	tRNA-Glu/23S ribosomal RNA
	pgap_annot_004004 → / → 004005	T→C	intergenic (+121/-64)	tRNA-Glu/23S ribosomal RNA
	pgap_annot_004004 → / → 004005	A→G	intergenic (+124/-61)	tRNA-Glu/23S ribosomal RNA
	pgap_annot_004004 → / → 004005	T→A	intergenic (+157/-28)	tRNA-Glu/23S ribosomal RNA
	mqo → / ← eco	G→A	intergenic (+425/+384)	malate dehydrogenase (quinone)/serine protease inhibitor ecotin
	mqo → / ← eco	A→G	intergenic (+430/+379)	malate dehydrogenase (quinone)/serine protease inhibitor ecotin
**Controls**	pgap_annot_001232 →	G→C	E252Q (GAA→CAA)	terminase ATPase subunit family protein
**Phage-selected**	waaA ← / → rfaQ	G→T	intergenic (-275/-138)	lipid IV(A) 3-deoxy-D-manno-octulosonic acid transferase/ lipopolysaccharide core heptosyltransferase RfaQ
	waaG →	Δ3 bp	coding (804-806/1125 nt)	glycosyltransferase family 4 protein
	waaG →	C→A	S339* (TCA→TAA)	glycosyltransferase family 4 protein
	[pgap_annot_003642–003643]	Δ777 bp		*
	[pgap_annot_003654–003666]	Δ7,259 bp		**
**excess-iron selected**	ydbD ←	G→T	L13I (CTT→ATT)	DUF2773 domain-containing bactofilin
	basR →	C→A	L99M (CTG→ATG)	two-component system response regulator
	fieF ← / ← cpxP	G→A	intergenic (-35/+114)	CDF family cation-efflux transporter FieF/cell-envelope stress modulator CpxP
	qseC ←	A→T	L299Q (CTG→CAG)	two-component system sensor histidine kinase QseC
	qseB ←	C→T	G15S (GGC→AGC)	two-component system response regulator QseB
	qseB ←	C→T	G15S (GGC→AGC)	two-component system response regulator QseB
**iron/phage-selected**	basR →	C→A	L99M (CTG→ATG)	two-component system response regulator BasR
	aroK →	C→T	Q23* (CAG→TAG)	shikimate kinase AroK
	rseB →	T→C	I308T (ATT→ACT)	sigma-E factor regulatory protein RseB

* Genes deleted: IspD/TarI family cytidylyltransferase and IS1-like element IS1A family transposase

** Genes deleted: DUF4942 domain-containing protein, DUF957 domain-containing protein, DUF5983 family protein, TA system toxin CbtA family protein, type IV toxin-antitoxin system YeeU family, antitoxin of toxin-antitoxin stability system, DUF987 domain-containing protein, RadC family protein, antirestriction protein, DUF932 domain-containing protein, DUF905 family protein, IrmA family protein, hypothetical protein

Supplementary Table S5: ***E. coli*** **B ATCC 11303 Genomic compositions**

**All genes**	**4,494**
**Protein coding**	4,206
**RNA genes**	116
**rRNAs (5S, 16S, 23S)**	8, 7, 7
**tRNAs**	85
**ncRNAs**	9
**Pseudogenes**	172

Supplementary B

Supplementary Table S6: Univariate analysis of the paired populations to each tested heavy and antibiotics

Substance	Compared groups	Range of substance tested	concentration	population	interaction
**Silver nitrate**	**Control = Phage-selected**	0 mg/L - 1 mg/L	F = 1.802, p = 0.182	F = 76.874, p = <0.001	F = 10.338, p = 0.002
**Sulfanilamide**	**Ancestor > Control**	0 mg/L - 2500 mg/L	F = 23.848, p = <0.001	F = 20.617, p = <0.001	F = 2.231,p = 0.019
**Tetracycline**	**Control < Iron(III)-selected**	0 mg/L - 6 mg/L	F = 6.569, p = 0.011	F = 301.553, p = <0.001	F = 11.678, p = <0.001
**Tetracycline**	**Control < Phage/Iron(III)-selected**	0 mg/L - 6 mg/L	F = 22.847, p = <0.001	F = 279.821, p = <0.001	F = 6.428, p = <0.001
	**Ancestor < Control**				
**Tetracycline**		0 mg/L - 6 mg/L	F = 50.936, p = <0.001	F = 216.216, p = <0.001	F = 5.29, p = <0.001
**Silver nitrate**		0 mg/L - 1 mg/L	F = 44.539, p = <0.001	F = 226.597, p = <0.001	F = 71.835, p = <0.001
**Gallium**		0 mg/L - 1750 mg/L	F = 267.855	F = 934.158, p = <0.001	F = 17.74, p = <0.001
**Iron(III) Sulfate**		0 mg/L - 1750 mg/L	F = 53.099, p = <0.001	F = 436.212, p = <0.001	F = 6.324, p = <0.001
**Iron (II) sulfate**		0 mg/L - 1750 mg/L	F = 86.822, p = <0.001	F = 367.054, p = <0.001	F = 5.788, p = <0.001
**copper (II) sulfate**		0 mg/L - 2500 mg/L	F = 0, p = 0.989	F = 469.331, p = <0.001	F = 5.562, p = <0.001
**Chloramphenicol**		0 mg/L - 1 mg/L	F = 12.63, p = 0.001	F = 579.017, p = <0.001	F = 12.63, p = 0.001
**Ampicillin**		2 mg/L - 4 mg/L	F = 17.775, p = <0.001	F = 168.778, p = <0.001	F = 1.625, p = 0.184
	**Ancestor > Phage-selected**				
**Sulfanilamide**		0 mg/L - 2500 mg/L	F = 23.831, p = <0.001	F = 16.374, p = <0.001	F = 2.024, p = 0.035
**Iron(III) Sulfate**		0 mg/L - 1750 mg/L	F = 55.404, p = <0.001	F = 172.029, p = <0.001	F = 4.345, p = <0.001
**Iron(II) sulfate**		0 mg/L - 1750 mg/L	F = 39.897, p = <0.001	F = 183.926, p = <0.001	F = 5.38, p = <0.001
	**Ancestor = Phage-selected**				
**copper (II) sulfate**		0 mg/L - 2500 mg/L	F = 44.326, p = <0.001	F = 321.256, p = <0.001	F = 2.496, p = 0.008
**Chloramphenicol**		0 mg/L - 1 mg/L	F = 0.388, p =0.534	F = 210.897, , p = <0.001	F = 0.388, p = 0.534
**Gallium**		0 mg/L - 1750 mg/L	F = 1.785, P = 0.182	F = 302.271, p = <0.001	F = 1.342, p = 0.22
	**Ancestor < Phage-selected**				
**Tetracycline**		0 mg/L - 6 mg/L	F = 149.92, p = <0.001	F = 127.477, p = <0.001	F = 21.654, p = <0.001
**Silver nitrate**		0 mg/L - 1 mg/L	F = 42.507p = <0.001	F = 585.244, p = <0.001	F = 35.822, p = <0.001
**Ampicillin**		2 mg/L - 4 mg/L	F = 84.755, p = <0.001	F = 116.607, p = <0.001	F = 15.543, p = <0.001
	**Ancestor < Iron(III)-selected**				
**Tetracycline**		0 mg/L - 6 mg/L	F = 94.722, p = <0.001	F = 316.671, p = <0.001	F = 36.289, p = <0.001
**Sulfanilamide**		0 mg/L - 2500 mg/L	F = 30.634, p = <0.001	F = 74.619, p = <0.001	F = 3.469, p = <0.001
**Silver nitrate**		0 mg/L - 1 mg/L	F = 28.706, p = <0.001	F = 1612, p = <0001	F = 28.706, p = <0.001
**Iron(III) Sulfate**		0 mg/L - 1750 mg/L	F = 197.34, p = <0.001	F = 194.062, p = <0.001	F = 32.402, p = <0.001
**Chloramphenicol**		0 mg/L - 1 mg/L	F = 17.069, , p = <0.001	F = 653.757, p = <0.001	F = 17.069, p = <0.001
**Ampicillin**		2 mg/L - 4 mg/L	F = 74.936, p = <0.001	F = 486.569, p = <0.001	F = 57.901, p = <0.001
	**Ancestor = Iron(III)-selected**				
**Gallium**		0 mg/L - 1750 mg/L	F = 2.532, p = 0.112	F = 261.291, p = <0.001	F = 26.647, p = <0.001
**Iron(II) sulfate**		0 mg/L - 1750 mg/L	F = 0.403, p = 0.526	F = 166.752, p = <0.001	F = 26.992, p = <0.001
**copper (II) sulfate**		0 mg/L - 2500 mg/L	F = 150.075, p = <0.001	F = 301.268, p = <0.001	F = 8.759, p = <0.001
	**Ancestor > Phage/Iron(III)-selected**				
**Sulfanilamide**		0 mg/L - 2500 mg/L	F = 26.201, p = <0.001	F = 83.806, p = <0.001	F = 5.965, p = <0.001
**Silver nitrate**		0 mg/L - 1 mg/L	F = 35.392, p = <0.001	F = 2011, p = <0.001	F = 35.392, p = <0.001
**Iron(III) Sulfate**		0 mg/L - 1750 mg/L	F = 124.086, p = <0.001	F = 206.075, p = <0.001	F = 27.927, p = <0.001
**Iron(II) sulfate**		0 mg/L - 1750 mg/L	F = 3.799, p = 0.052	F = 108.205, p = <0.001	F = 35.028, p = <0.001
	**Ancestor < Phage/Iron(III)-selected**				
**Tetracycline**		0 mg/L - 6 mg/L	F = 168.625, p = <0.001	F = 297.953, p = <0.001	F = 25.021, p = <0.001
**Gallium**		0 mg/L - 1750 mg/L	F = 13.726, p = <0.001	F = 325.921, p = <0.001	F = 27.168, p = <0.001
**copper (II) sulfate**		0 mg/L - 2500 mg/L	F = 31.204, p = <0.001	F = 351.597, p = <0.001	F = 3.539, p = <0.001
**Chloramphenicol**		0 mg/L - 1 mg/L	F = 22.934, , p = <0.001	F = 788.891, p = <0.001	F = 22.934, p = <0.001
**Ampicillin**		2 mg/L - 4 mg/L	F = 61.155, p = <0.001	F = 373.981, p = <0.001	F = 27.857, p = <0.001

Substance	Compared groups	Range of substance tested	concentration	population	interaction
	**Control < Phage-selected**				
**Tetracycline**		0 mg/L - 6 mg/L	F = 64.367, p = <0.001	F = 142.107, p = <0.001	F = 10.689, p = <0.001
**Ampicillin**		2 mg/L - 4 mg/L	F = 40.703, p = <0.001	F = 118.801, p = <0.001	F = 9.251, p = <0.001
	**Control > Phage-selected**				
**Sulfanilamide**		0 mg/L - 2500 mg/L	F = 0.21, p = 0.647	F = 7.403, p = <0.001	F = 0.17, p = 0.997
**Gallium**		0 mg/L - 1750 mg/L	F = 103.527, p = <0.001	F = 470.12, p = <0.001	F = 9.413, p = <0.001
**Iron(III) Sulfate**		0 mg/L - 1750 mg/L	F = 278.261, p = <0.001	F = 339.954, p = <0.001	F = 16.501, p = <0.001
**Iron(II) sulfate**		0 mg/L - 1750 mg/L	F = 254.017, p = <0.001	F = 258.378, p = <0.001	F = 10.759, p = <0.001
**copper (III) sulfate**		0 mg/L - 2500 mg/L	F = 73.35, p = <0.001	F = 561.642, p = <0.001	F = 12.172, p = <0.001
**Chloramphenicol**		0 mg/L - 1 mg/L	F = 13.277, , p = <0.001	F = 370.094, p = <0.001	F = 13.277, p = <0.001
	**Control > Iron(III)-selected**				
**Silver nitrate**		0 mg/L - 1 mg/L	F = 83.922, p = <0.001	F = 141.954, p = <0.001	F = 30.626, p = <0.001
**Gallium**		0 mg/L - 1750 mg/L	F = 238.765, p = <0.001	F = 450.33, p = <0.001	F = 76.927, p = <0.001
**Iron(III) Sulfate**		0 mg/L - 1750 mg/L	F = 1342, p = <0.001	F = 715.263, p = <0.001	F = 158.358, p = <0.001
**Iron(II) sulfate**		0 mg/L - 1750 mg/L	F = 80.239, p = <0.001	F = 252.146, p = <0.001	F = 38.631, p = <0.001
**copper (III) sulfate**		0 mg/L - 2500 mg/L	F = 263.953, p = <0.001	F = 563.266, p = <0.001	F = 24.768, p = <0.001
	**Control = Iron(III)-selected**				
**Sulfanilamide**		0 mg/L - 2500 mg/L	F = 0.764, p = 0.383	F = 35.126, p = <0.001	F = 7.904, p = <0.001
**Chloramphenicol**		0 mg/L - 1 mg/L	F = 0.295, p = 0.588	F = 1458, p = <0.001	F = 0.295, p = 0.588
**Ampicillin**		2 mg/L - 4 mg/L	F = 7.914, p = 0.005	F = 364.875, p = <0.001	F = 24.707, p = <0.001
	**Control = Phage/Iron(III)-selected**				
**Sulfanilamide**		0 mg/L - 2500 mg/L	F = 2.851, p = 0.092	F = 37.899, p = <0.001	F = 9.111, p = <0.001
**Chloramphenicol**		0 mg/L - 1 mg/L	F = 1.101, p = 0.296	F = 2010, p = <0.001	F = 1.101, p = 0.296
**Ampicillin**		2 mg/L - 4 mg/L	F = 5.116, p =0.025	F = 289.318, p = <0.001	F = 10.147, p = <0.001
	**Control > Phage/Iron(III)-selected**				
**Silver nitrate**		0 mg/L - 1 mg/L	F = 88.035, p = <0.001	F = 149.736, p = <0.001	F = 32.394, p = <0.001
**Gallium**		0 mg/L - 1750 mg/L	F = 117.938, p = <0.001	F = 580.198, p = <0.001	F = 84.506, p = <0.001
**Iron(III) Sulfate**		0 mg/L - 1750 mg/L	F = 1013, p = <0.001	F = 760.961, p = <0.001	F = 145.563, p = <0.001
**Iron(II) sulfate**		0 mg/L - 1750 mg/L	F = 117.445, p = <0.001	F = 160.611, p = <0.001	F = 47.606, p = <0.001
**copper (III) sulfate**		0 mg/L - 2500 mg/L	F = 53.669, p = <0.001	F = 643.369, p = <0.001	F = 9.983, p = <0.001
	**Phage-selected < Iron(III)-selected**				
**Sulfanilamide**		0 mg/L - 2500 mg/L	F = 1.723, p = 0.19	F = 26.97, p = <0.001	F = 5.463, p = <0.001
**Iron(II) sulfate**		0 mg/L - 1750 mg/L	F = 50.394, p = <0.001	F = 112.894, p = <0.001	F = 8.719, p = <0.001
**Chloramphenicol**		0 mg/L - 1 mg/L	F = 16.768, p = <0.001	F = 406.561, p = <0.001	F = 16.768, p = <0.001
	**Phage-selected > Iron(III)-selected**				
**Tetracycline**		0 mg/L - 6 mg/L	F = 0, p = 1	F = 139.903, p = <0.001	F = 0, p = 1
**Silver nitrate**		0 mg/L - 1 mg/L	F = 91.966, p = <0.001	F = 386.08, p = <0.001	F = 5.361, p = 0.022
**Gallium**		0 mg/L - 1750 mg/L	F = 6.282, p = 0.012	F = 170.222, p = <0.001	F = 15.22, p = <0.001
**Iron(III) Sulfate**		0 mg/L - 1750 mg/L	F = 18.303, p = <0.001	F = 136.87, p = <0.001	F = 12.799, p = <0.001
**copper (III) sulfate**		0 mg/L - 2500 mg/L	F = 36.811, p = <0.001	F = 340.703, p = <0.001	F = 3.411, p = <0.001
**Ampicillin**		2 mg/L - 4 mg/L	F = 25.924, p = <0.001	F = 206.967, p = <0.001	F = 0.291, p = 0.832
	**Phage-selected = Phage/Iron(III)-selected**				
**copper (III) sulfate**		0 mg/L - 2500 mg/L	F = 2.07, p = 0.151	F = 400.431, p = <0.001	F = 0.974, p = 0.46
**Gallium**		0 mg/L - 1750 mg/L	F = 2.577, p = 0.109	F = 200.237, p = <0.001	F = 14.476, p = <0.001
	**Phage-selected < Phage/Iron(III)-selected**				
**Sulfanilamide**		0 mg/L - 2500 mg/L	F = 4.097, p = 0.043	F = 28.685, p = <0.001	F = 5.656, p = <0.001
**Iron(II) sulfate**		0 mg/L - 1750 mg/L	F = 16.874, p = <0.001	F = 71.675, p = <0.001	F = 13.741, p = <0.001
**Chloramphenicol**		0 mg/L - 1 mg/L	F = 20.785, , p = <0.001	F = 463.048, p = <0.001	F = 20.785, p = <0.001

Substance	Compared groups	Range of substance tested	concentration	population	interaction
Ampicillin	**Phage-selected Phage/Iron(III)-selected**	2 mg/L - 4 mg/L	F = 28.725, p = <0.001	F = 175.632, p = <0.001	F = 2.225, p = 0.086
Tetracycline		0 mg/L - 6 mg/L	F = 0, p = 1	F = 139.903, p = <0.001	F = 0, p = 1
Silver nitrate		0 mg/L - 1 mg/L	F = 99.451, p = <0.001	F = 419.848, p = <0.001	F = 5.91, p = 0.017
Iron(III) Sulfate		0 mg/L - 1750 mg/L	F = 1.965, p = 0.162,	F = 145.746, p = <0.001	F = 10.77, p = <0.001
	**Iron(III)-selected = Phage/Iron(III)-selected**				
Tetracycline		0 mg/L - 6 mg/L	F = 3.9, p = 0.049	F = 377.364, p = <0.001	F = 8.164, p = <0.001
Sulfanilamide		0 mg/L - 2500 mg/L	F = 1.81, p = 0.179	F = 246.248, p = <0.001	F = 1.551, p = 0.127
Silver nitrate		0 mg/L - 1 mg/L	F = 0.001, p = 0.978	F = 1169, p = <0.001	F = 0.001, p = 0.978
Chloramphenicol		0 mg/L - 1 mg/L	F = 0.221, p = 0.639	F = 2626, p = <0.001	F = 0.221, p =0.639
Ampicillin		2 mg/L - 4 mg/L	F = 44.7, p = <0.001	F = 148.457, p = <0.001	F = 9.892, p = <0.001
	**Iron(III)-selected < Phage/Iron(III)-selected**				
Gallium		0 mg/L - 1750 mg/L	F = 22.952, p = <0.001	F = 174.091, p = <0.001	F = 1.574, p = 0.13
Iron(III) Sulfate		0 mg/L - 1750 mg/L	F = 25.177, p = <0.001	F = 261.353, p = <0.001	F = 1.149, p = 0.331
Iron(II) sulfate		0 mg/L - 1750 mg/L	F = 6.749, p = <0.001	F = 72.822, p = <0.001	F = 2.633, p = 0.008
copper (III) sulfate		0 mg/L - 2500 mg/L	F = 61.555, p = <0.001	F = 386.84, p = <0.001	F = 4.7, p = <0.001

Supplementary B

Supplementary Table S7 : Bonferroni's multiple comparisons of cross-resistance testing of Ancestor, Control, Phage-selected, Fe(III)-selected, and Fe(III)-phage-selected in heavy metals

					95% Confidence Interval	
**Substance**	**(I) Population**	**(J) Population**	**Mean Difference (I-J)**	**Sig.**	**Lower Bound**	**Upper Bound**
**Copper (II) sulfate**	**Ancestor**	control	-.0786*	.000	-.0972	-.0601
		phage	-.0086	1.000	-.0272	.0100
		Fe(III)-selected	.0115	.806	-.0070	.0301
		Phage/Iron(III)-selected	-.0211*	.014	-.0397	-.0025
	**control**	phage	.0700*	.000	.0514	.0886
		Fe(III)-selected	.0902*	.000	.0716	.1088
		Phage/Iron(III)-selected	.0575*	.000	.0389	.0761
	**phage**	Fe(III)-selected	.0202*	.023	.0016	.0387
		Phage/Iron(III)-selected	-.0125	.589	-.0311	.0061
	**Fe(III)-selected**	Phage/Iron(III)-selected	.0327*	.000	.0141	.0513
**Sulfanilamide**	**Ancestor**	control	.0590*	.000	.0248	.0932
		phage	.0869*	.000	.0507	.1230
		Fe(III)-selected	.0573*	.000	.0222	.0924
		Phage/Iron(III)-selected	.0488*	.001	.0137	.0839
	**control**	phage	.0278	.269	-.0075	.0631
		Fe(III)-selected	-.0017	1.000	-.0360	.0325
		Phage/Iron(III)-selected	-.0102	1.000	-.0444	.0240
	**phage**	Fe(III)-selected	-.0296	.216	-.0657	.0066
		Phage/Iron(III)-selected	-.0380*	.031	-.0742	-.0019
	**Fe(III)-selected**	Phage/Iron(III)-selected	-.0085	1.000	-.0436	.0266
**Silver nitrate**	**Ancestor**	control	-.1877*	.000	-.2548	-.1205
		phage	-.1429*	.000	-.2101	-.0758
		Fe(III)-selected	.0779*	.012	.0107	.1451
		Phage/Iron(III)-selected	.0775*	.012	.0103	.1447
	**control**	phage	.0447	.607	-.0225	.1119
		Fe(III)-selected	.2656*	.000	.1984	.3327
		Phage/Iron(III)-selected	.2652*	.000	.1980	.3323
	**phage**	Fe(III)-selected	.2208*	.000	.1537	.2880
		Phage/Iron(III)-selected	.2204*	.000	.1533	.2876
	**Fe(III)-selected**	Phage/Iron(III)-selected	-.0004	1.000	-.0676	.0668
**Iron(II) Sulfate**	**Ancestor**	control	-.0790*	.000	-.1057	-.0524
		phage	.0633*	.000	.0367	.0900
		Fe(III)-selected	-.0059	1.000	-.0326	.0207
		Phage/Iron(III)-selected	.0199	.362	-.0068	.0466
	**control**	phage	.1424*	.000	.1157	.1691
		Fe(III)-selected	.0731*	.000	.0464	.0998
		Phage/Iron(III)-selected	.0989*	.000	.0723	.1256
	**phage**	Fe(III)-selected	-.0693*	.000	-.0959	-.0426
		Phage/Iron(III)-selected	-.0435*	.000	-.0701	-.0168
	**Fe(III)-selected**	Phage/Iron(III)-selected	.0258	.066	-.0009	.0525
**Gallium Nitrate**	**Ancestor**	control	-.0884*	.000	-.1091	-.0677
		phage	-.0110	1.000	-.0317	.0096
		Fe(III)-selected	.0115	1.000	-.0092	.0322
		Phage/Iron(III)-selected	-.0248*	.008	-.0455	-.0041
	**control**	phage	.0774*	.000	.0567	.0980
		Fe(III)-selected	.0999*	.000	.0792	.1206
		Phage/Iron(III)-selected	.0636*	.000	.0429	.0843
	**phage**	Fe(III)-selected	.0225*	.022	.0019	.0432
		Phage/Iron(III)-selected	-.0138	.615	-.0344	.0069
	Fe(III)-selected	Phage/Iron(III)-selected	.0363*	.000	.0156	.0570

Based on observed means.
*. The mean difference is significant at the 0.05 level.

Supplementary Table S8: Bonferroni's multiple comparisons of cross-resistance testing of Ancestor, Control, Phage-selected, Fe(III)-selected, and Fe(III)-phage-selected in Antibiotics

					95% Confidence Interval	
**Substance**	**(I) Population**	**(J) Population**	**Mean Difference (I-J)**	**Sig.**	**Lower Bound**	**Upper Bound**
**Ampicillin**	**Ancestor**	control	-.0558*	.014	-.1047	-.0068
		phage	-.2086*	.000	-.2576	-.1597
		Fe(III)-selected	-.0931*	.000	-.1421	-.0442
		Phage/Iron(III)-selected	-.0863*	.000	-.1353	-.0374
	**control**	phage	-.1529*	.000	-.2018	-.1039
		Fe(III)-selected	-.0373	.320	-.0863	.0116
		Phage/Iron(III)-selected	-.0305	.792	-.0795	.0184
	**phage**	Fe(III)-selected	.1155*	.000	.0666	.1645
		Phage/Iron(III)-selected	.1223*	.000	.0734	.1713
	**Fe(III)-selected**	Phage/Iron(III)-selected	-.0068	1.000	-.0557	.0421
**Tetracycline**	**Ancestor**	control	-.0691*	.000	-.1098	-.0285
		phage	-.2214*	.000	-.2621	-.1807
		Fe(III)-selected	-.0991*	.000	-.1398	-.0585
		Phage/Iron(III)-selected	-.1217*	.000	-.1624	-.0811
	**control**	phage	-.1522*	.000	-.1929	-.1116
		Fe(III)-selected	-.0300	.382	-.0707	.0107
		Phage/Iron(III)-selected	-.0526*	.003	-.0932	-.0119
	**phage**	Fe(III)-selected	.1223*	.000	.0816	.1629
		Phage/Iron(III)-selected	.0997*	.000	.0590	.1403
	**Fe(III)-selected**	Phage/Iron(III)-selected	.0226	1.000	-.0181	.0633
**Chloramphenicol**	**Ancestor**	control	-.0629*	.004	-.1126	-.0133
		phage	.0149	1.000	-.0347	.0646
		Fe(III)-selected	-.0700*	.001	-.1197	-.0203
		Phage/Iron(III)-selected	-.0747*	.000	-.1243	-.0250
	**control**	phage	.0779*	.000	.0282	.1276
		Fe(III)-selected	-.0071	1.000	-.0567	.0426
		Phage/Iron(III)-selected	-.0117	1.000	-.0614	.0380
	**phage**	Fe(III)-selected	-.0849*	.000	-.1346	-.0353
		Phage/Iron(III)-selected	-.0896*	.000	-.1393	-.0399
	**Fe(III)-selected**	Phage/Iron(III)-selected	-.0047	1.000	-.0543	.0450

Based on observed means.

*. The mean difference is significant at the 0.05 level.
